# 1184. A Phase 3, Multicenter, Randomized, Double-blind Study to Evaluate the Interchangeability of V114 and Prevnar 13™ with Respect to Safety, Tolerability, and Immunogenicity in Healthy Infants (PNEU-DIRECTION)

**DOI:** 10.1093/ofid/ofab466.1376

**Published:** 2021-12-04

**Authors:** Adroniki Bili, Scott Dobson, Jeffrey Quinones, Wanatpreeya Phongsamart, Peninnah Oberdorfer, Pope Kosalaraksa, Ron Dagan, Marissa B Wilck, Waldimir Vallejos, Christine Nunn, Richard McFetridge, Rong Fu, Robert Lupinacci, Luwy Musey, Kara Bickham

**Affiliations:** 1 Merck & Co., Inc., Kenilworth, New Jersey; 2 Parkside Clinical Research and Tribe Clinical Research, Greenville, South Carolina; 3 Clinical Research of Puerto Rico, Guayama, Puerto Rico; 4 Mahidol University, Bangkok, Krung Thep, Thailand; 5 Chiang Mai University, Chiang Mai, Chiang Mai, Thailand; 6 Khon Kaen University, Khon Kaen, Khon Kaen, Thailand; 7 Ben-Gurion University of the Negev, Beer Sheva, HaDarom, Israel; 8 MSD China, Shangai, Shanghai, China

## Abstract

**Background:**

Pneumococcal diseases (PD) caused by *Streptococcus pneumoniae* are a major health concern globally. In children, currently licensed pneumococcal conjugate vaccines (PCVs) provide protection against PD from vaccine serotypes, but other non-vaccine serotypes have emerged and contribute to most residual disease. V114 is a 15-valent investigational PCV containing serotypes 22F and 33F in addition to the 13 serotypes shared by Prevnar 13^TM^ (PCV13). This phase 3 study evaluated safety and immunogenicity of mixed PCV13/V114 regimens when changing from PCV13 to V114 at doses 2, 3, or 4.

**Methods:**

In this double-blind trial, 900 infants were randomized in equal ratios to five treatment groups using a 3 + 1 immunization schedule (3-dose infant primary series followed by one toddler dose). Groups 2, 3, and 4 started with PCV13 and switched to V114 at doses 4, 3, and 2, respectively. Groups 1 and 5 received four doses of PCV13 and V114, respectively. Immunoglobulin G (IgG) responses to the 15 pneumococcal serotypes in V114 were measured at 30 days post-dose 3, prior to dose 4, and 30 days post-dose 4 (PD4). Primary immunogenicity analysis was based on 13 shared serotype responses at PD4. Safety was evaluated as the proportion of participants with adverse events (AEs).

**Results:**

At 30 days PD4, IgG geometric mean concentrations (GMCs) for the 13 shared serotypes were generally comparable between V114/PCV13 mixed regimens (Groups 2-4) and participants that received the 4-dose PCV13 regimen (Group 1). Additionally, IgG GMCs for the 13 shared serotypes were generally comparable for participants that received the 4-dose V114 regimen (Group 5) and participants that received the 4-dose PCV13 regimen (Group 1). Infants given at least one dose of V114 mounted immune responses to two unique serotypes in V114 (22F and 33F). Frequency of injection-site and systemic AEs among study participants were generally comparable across all study groups.

**Conclusion:**

V114 was well tolerated with a generally comparable safety profile to PCV13. For the 13 shared serotypes, both mixed-dose and 4-dose regimens of V114 induced generally comparable antibody responses to a PCV13 4-dose regimen. Study results support interchangeability of V114 with PCV13 in infants.

**Disclosures:**

**Adroniki Bili, MD**, **Merck & Co., Inc.** (Employee, Shareholder) **Ron Dagan, MD**, **Medimmune/AstraZeneca** (Grant/Research Support, Scientific Research Study Investigator, Research Grant or Support)**MSD** (Consultant, Grant/Research Support, Scientific Research Study Investigator, Advisor or Review Panel member, Research Grant or Support, Speaker’s Bureau)**Pfizer** (Consultant, Grant/Research Support, Scientific Research Study Investigator, Advisor or Review Panel member, Research Grant or Support, Speaker’s Bureau) **Marissa B. Wilck, MD**, **Merck & Co., Inc.** (Employee, Shareholder) **Waldimir Vallejos, MD**, **Merck & Co., Inc.** (Employee, Shareholder) **Christine Nunn, MS**, **Merck & Co., Inc.** (Employee, Shareholder) **Richard McFetridge, B.S.**, **Merck & Co., Inc** (Employee) **Rong Fu, PhD**, **MSD China** (Employee, Shareholder) **Robert Lupinacci, M.S**, **Merck & Co., Inc** (Employee, Shareholder) **Luwy Musey, MD**, **Merck & Co., Inc.** (Employee) **Kara Bickham, MD**, **Merck Sharp and Dohme** (Employee, Shareholder)

